# Supplementary material for: Semaphorin 3A: A potential target for prevention and treatment of nickel allergy
Source: Commun Biol. 2022 Jul 7;5:671. doi: 10.1038/s42003-022-03641-0 (PMC9262932; doi:10.1038/s42003-022-03641-0)
Supplement: Supplementary file 2 — Supplementary Information [file 42003_2022_3641_MOESM2_ESM.pdf]

Supplementary Information

Semaphorin 3A: A Potential Target for Prevention and Treatment of Nickel Allergy

Lipei Liu[1], Megumi Watanabe[1]*, Norikazu Minami[1], Mohammad Fadyl Yunizar[1], Tetsuo Ichikawa[1]

[1] Department of Prosthodontics & Oral Rehabilitation, Tokushima University, Graduate School of Biomedical Sciences, 3-18-15, Kuramoto, Tokushima, 770-8504, Japan.

* Correspondence to Megumi Watanabe.

Email: megwat@tokushima-u.ac.jp

Supplementary Information includes:

Supplementary Figure 1. The extraction example of Sema3A-positive area in Pam2.12 after Ni stimulation.

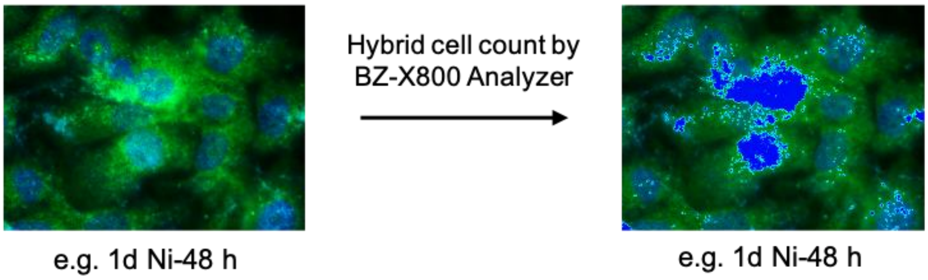

The example of fluorescence hybrid cell count by BZ-X800 Analyzer software was given in which Sema3A-positive area will be extracted as blue color.

Supplementary Figure 2. The extraction example of Sema3A-positive area in the total area of epidermis in ear tissue.

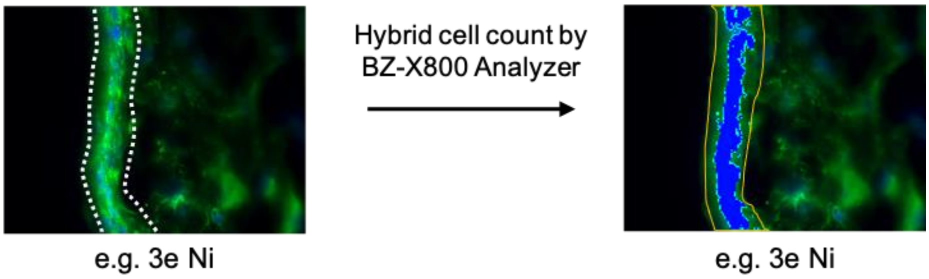

The example of fluorescence hybrid cell count by BZ-X800 Analyzer software was given in which Sema3A-positive area will be extracted as blue color and the yellow line indicates the specified area of epidermis.

Supplementary Figure 3. The extraction example of double-positive area in the immune cells in the total area of ear tissue.

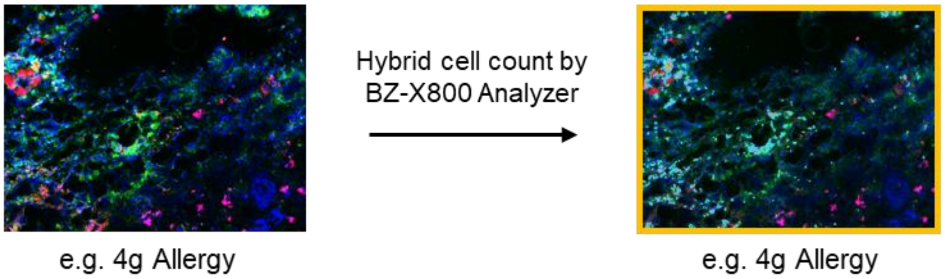

The example of fluorescence hybrid cell count by BZ-X800 Analyzer software was given in which double positive area will be extracted as pink color and the yellow line indicates the specified area of ear tissue.

Supplementary Figure 4. Ni allergy induction in mice.

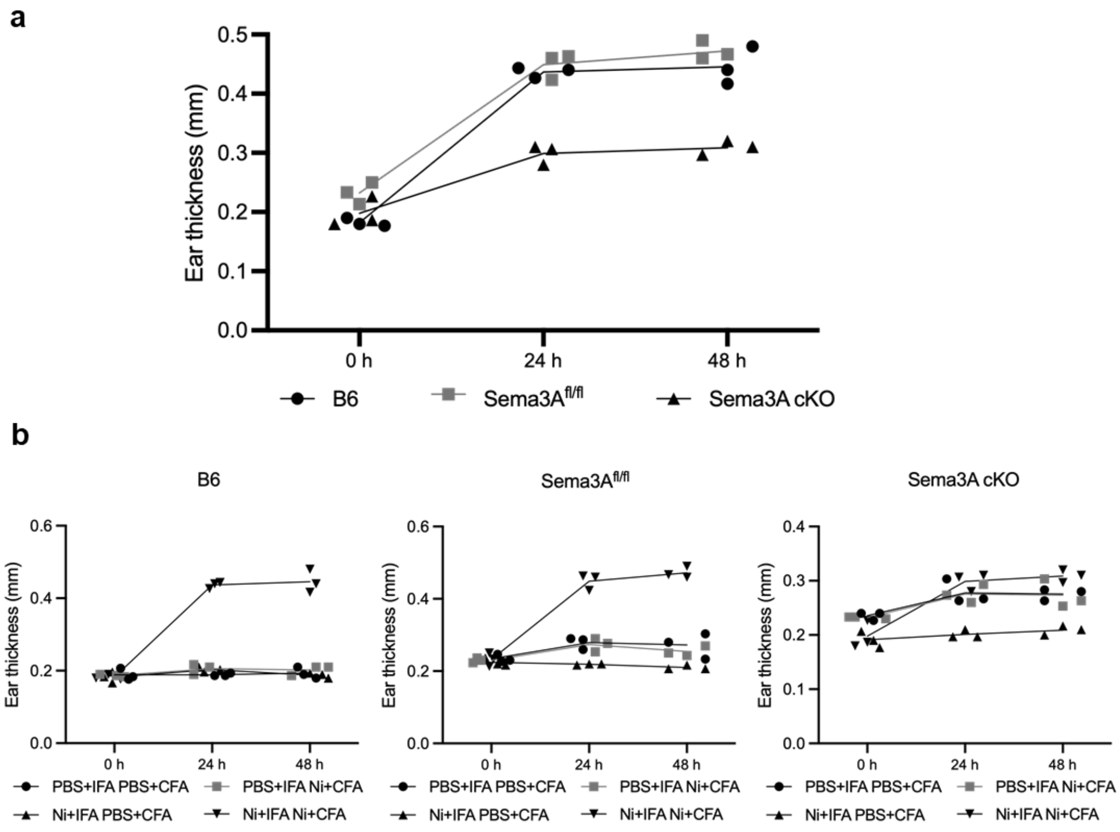

a Ni allergy could be induced in Sema3A$^{fl/fl}$ mice similarly to that in C57BL/6J mice. DTH was determined by ear thickness measuring after Ni re-challenge. The ear thickness change of three kinds of mice has been shown (N=3). Supplementary data and p values are provided as a Supplementary Data file. **b** Ear thickness was not increased in unsensitized mice. For initial immunization, $NiCl_2$ with IFA (sensitized) or PBS with IFA (unsensitized) was intraperitoneally injected to mice. For re-challenge, $NiCl_2$ with CFA or PBS with CFA was intradermally injected into the ear skin 2 weeks later. DTH was determined by ear thickness measuring after Ni re-challenge. The ear thickness increased only in the Ni-injected ear tissue of sensitized mice. N=3. Supplementary data and p values are provided as a Supplementary Data file.

Supplementary Figure 5. Uncropped Western blotting images.

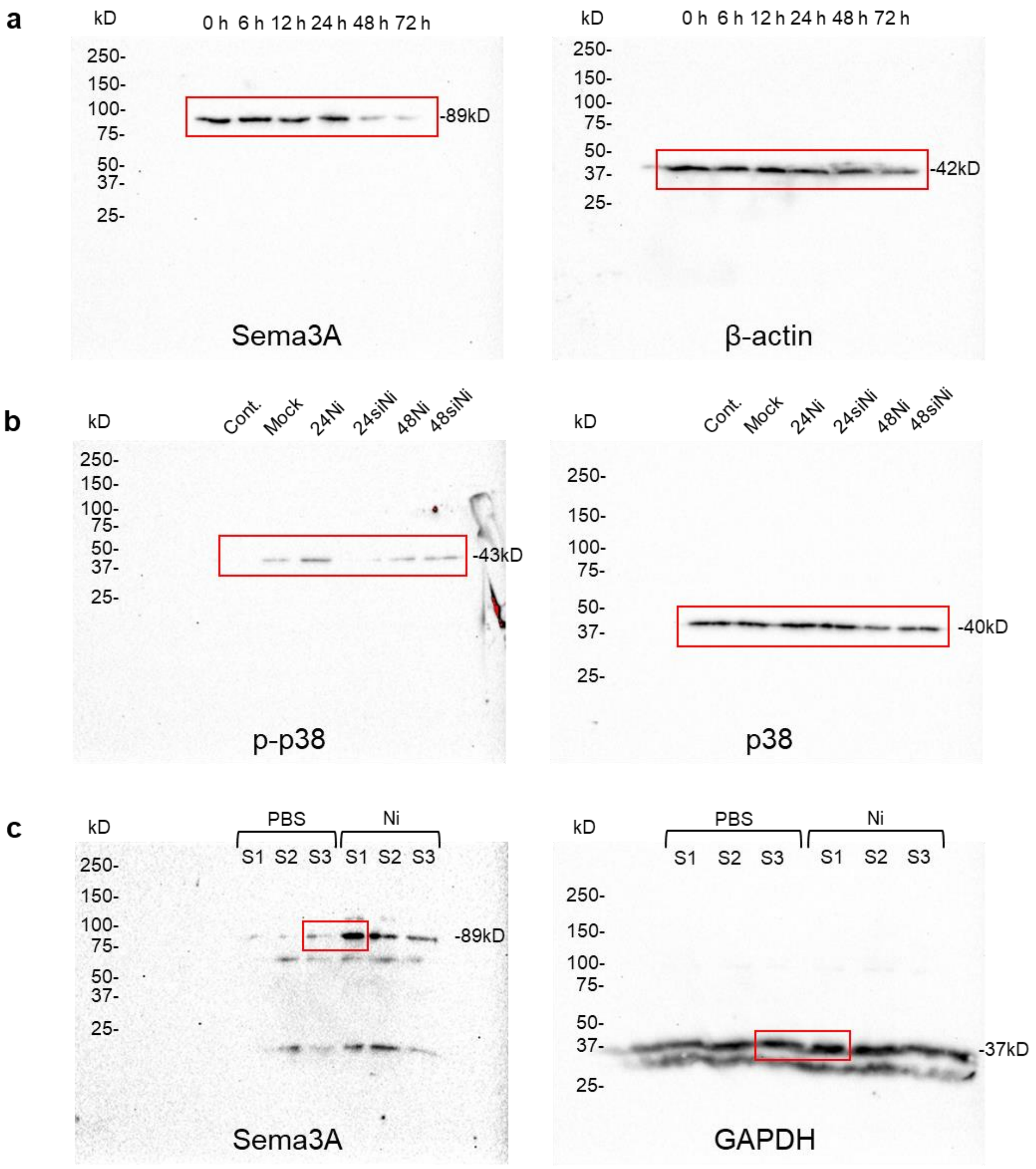

Original uncropped Western blots used for Figure 1b (**a**)，2c (**b**) and 3f (**c**) are shown.

Red frames show the protein bands used in the figure.
