# Supplementary material for: Semaphorin 3A: A potential target for prevention and treatment of nickel allergy
Source: Commun Biol. 2022 Jul 7;5:671. doi: 10.1038/s42003-022-03641-0 (PMC9262932; doi:10.1038/s42003-022-03641-0)
Supplement: Supplementary file 3 — Description of Additional Supplementary Files [file 42003_2022_3641_MOESM3_ESM.pdf]

Description of Additional Supplementary Files

File name: Supplementary Data
Description: The source data behind the graphs in the paper.
