# Supplementary material for: Semaphorin 3A: A potential target for prevention and treatment of nickel allergy
Source: Commun Biol. 2022 Jul 7;5:671. doi: 10.1038/s42003-022-03641-0 (PMC9262932; doi:10.1038/s42003-022-03641-0)
Supplement: Supplementary file 5 — Reporting Summary [file 42003_2022_3641_MOESM5_ESM.pdf]

nature portfolio

Corresponding author(s): Megumi Watanabe

Last updated by author(s): May 26, 2022

Reporting Summary

Nature Portfolio wishes to improve the reproducibility of the work that we publish. This form provides structure for consistency and transparency in reporting. For further information on Nature Portfolio policies, see our Editorial Policies and the Editorial Policy Checklist.

Statistics

For all statistical analyses, confirm that the following items are present in the figure legend, table legend, main text, or Methods section.

n/a	Confirmed	
☐	☒	The exact sample size (n) for each experimental group/condition, given as a discrete number and unit of measurement
☐	☒	A statement on whether measurements were taken from distinct samples or whether the same sample was measured repeatedly
☐	☒	The statistical test(s) used AND whether they are one- or two-sided<br>*Only common tests should be described solely by name; describe more complex techniques in the Methods section.*
☒	☐	A description of all covariates tested
☒	☐	A description of any assumptions or corrections, such as tests of normality and adjustment for multiple comparisons
☐	☒	A full description of the statistical parameters including central tendency (e.g. means) or other basic estimates (e.g. regression coefficient) AND variation (e.g. standard deviation) or associated estimates of uncertainty (e.g. confidence intervals)
☒	☐	For null hypothesis testing, the test statistic (e.g. F, t, r) with confidence intervals, effect sizes, degrees of freedom and P value noted<br>*Give P values as exact values whenever suitable.*
☒	☐	For Bayesian analysis, information on the choice of priors and Markov chain Monte Carlo settings
☒	☐	For hierarchical and complex designs, identification of the appropriate level for tests and full reporting of outcomes
☒	☐	Estimates of effect sizes (e.g. Cohen's d, Pearson's r), indicating how they were calculated

Our web collection on statistics for biologists contains articles on many of the points above.

Software and code

Policy information about availability of computer code

Data collection	Quantitative RT-PCR data were collected using ChemiDoc XRS (Bio-Rad, Hercules, CA, USA).<br>Western blotting data were collected using ABI7300 Real-time PCR System (Applied Biosystems, MA, USA).<br>Immunofluorescence images were captured using Keyence all-in-one fluorescence-microscope BZ-X800 (KEYENCE CORPORATION, Tokyo, Japan).<br>Flow cytometry data were collected using BD FACSVerse™ (BDBiosciences, San Jose, CA, USA).
Data analysis	Image Lab was used for western blot analysis.<br>7300 System SDS Software was used for qRT-PCR analysis.<br>BZ-X800 Analyzer was used for immunofluorescence image treatment.<br>BD FACSuite Software was used for FACS analysis.

For manuscripts utilizing custom algorithms or software that are central to the research but not yet described in published literature, software must be made available to editors and reviewers. We strongly encourage code deposition in a community repository (e.g. GitHub). See the Nature Portfolio guidelines for submitting code & software for further information.

Data

Policy information about availability of data

All manuscripts must include a data availability statement. This statement should provide the following information, where applicable:

- Accession codes, unique identifiers, or web links for publicly available datasets
- A description of any restrictions on data availability
- For clinical datasets or third party data, please ensure that the statement adheres to our policy

All the other data are available in the main text. The raw data that support all the remaining figures are provided in the Supplementary Data File.

Human research participants

Policy information about studies involving human research participants and Sex and Gender in Research.

Reporting on sex and gender	*Use the terms sex (biological attribute) and gender (shaped by social and cultural circumstances) carefully in order to avoid confusing both terms. Indicate if findings apply to only one sex or gender; describe whether sex and gender were considered in study design whether sex and/or gender was determined based on self-reporting or assigned and methods used. Provide in the source data disaggregated sex and gender data where this information has been collected, and consent has been obtained for sharing of individual-level data; provide overall numbers in this Reporting Summary. Please state if this information has not been collected. Report sex- and gender-based analyses where performed, justify reasons for lack of sex- and gender-based analysis.*
Population characteristics	*Describe the covariate-relevant population characteristics of the human research participants (e.g. age, genotypic information, past and current diagnosis and treatment categories). If you filled out the behavioural & social sciences study design questions and have nothing to add here, write "See above."*
Recruitment	*Describe how participants were recruited. Outline any potential self-selection bias or other biases that may be present and how these are likely to impact results.*
Ethics oversight	*Identify the organization(s) that approved the study protocol.*

Note that full information on the approval of the study protocol must also be provided in the manuscript.

Field-specific reporting

Please select the one below that is the best fit for your research. If you are not sure, read the appropriate sections before making your selection.

☒ Life sciences ☐ Behavioural & social sciences ☐ Ecological, evolutionary & environmental sciences

For a reference copy of the document with all sections, see nature.com/documents/nr-reporting-summary-flat.pdf

Life sciences study design

All studies must disclose on these points even when the disclosure is negative.

Sample size	For in vivo experiment in Figure 3 and Figure 4, there were 5 mice for each group.<br>For in vivo experiment in Figure 5, there were 3 mice for each group.<br>For in vivo experiment in Figure 6a, there were 4 mice for each group.<br>For in vivo experiment in Figure 6b and Figure 6c, there were 7 mice for each group.
Data exclusions	In most experiments no data was excluded. In few experiments outliers were excluded.
Replication	Results were reliably reproduced for each experiment. This included independent replication: all experiments presented in this study were performed using at least two biological replicates.
Randomization	Mice were randomly chosen amongst groups before induction of Ni allergy.
Blinding	The investigators performing qRT-PCR, immunofluorescence, FACS and analyses were blinded to group allocation.

Reporting for specific materials, systems and methods

We require information from authors about some types of materials, experimental systems and methods used in many studies. Here, indicate whether each material, system or method listed is relevant to your study. If you are not sure if a list item applies to your research, read the appropriate section before selecting a response.

Materials & experimental systems

n/a	Involved in the study
☐	☒ Antibodies
☐	☒ Eukaryotic cell lines
☒	☐ Palaeontology and archaeology
☐	☒ Animals and other organisms
☒	☐ Clinical data
☒	☐ Dual use research of concern

Methods

n/a	Involved in the study
☒	☐ ChIP-seq
☐	☒ Flow cytometry
☒	☐ MRI-based neuroimaging

Antibodies

Antibodies used

Anti-Semaphorin 3A antibody, Abcam, ab23393
Phospho-p38 MAPK (Thr180/Tyr182) Antibody, Cell Signaling, #9211
p38 MAPK Antibody, Cell Signaling, #9212
Rabbit antibody to GAPDH, Osenses, OSG00033G
beta Actin Polyclonal Antibody, Bioss, bs-0061R
Anti-rabbit IgG, HRP-linked antibody, Cell Signaling, #7074
Alexa Fluor 488 anti-mouse CD11c Antibody, BioLegend, 117311
Alexa Fluor 488 anti-mouse/human CD11b Antibody, BioLegend, 101217
PE anti-mouse F4/80 Antibody, BioLegend, 123110
PE anti-mouse I-A/I-E Antibody, BioLegend, 107608
FITC anti-mouse CD3 Antibody, BioLegend, 100203
PE/Cyanine7 anti-mouse CD4 Antibody, BioLegend, 100527
PE anti-mouse CD8a Antibody, BioLegend, 100707
APC-Cy™7 Rat Anti-Mouse CD45, BD Biosciences, 557659
Purified Rat Anti-Mouse CD16/CD32 (Mouse BD Fc Block™), BD Biosciences, 553142
eBioscience™ 7-AAD Viability Staining Solution, Invitrogen™, 00-6993-50
Donkey Anti-Rabbit IgG H&L (Alexa Fluor® 488), Abcam, ab150073

Validation

The antibodies were validated according to the manufacturer.

Eukaryotic cell lines

Policy information about cell lines and Sex and Gender in Research

Cell line source(s)

Mouse keratinocyte cell line Pam2.12 was kindly provided by Dr. S. H. Yuspa (National Cancer Institute, Bethesda).

Authentication

The cell line was not authenticated.

Mycoplasma contamination

Cells were negative for mycoplasma contamination.

Commonly misidentified lines
(See ICLAC register)

The cell line used in this study was not found in the database of commonly misidentified cell lines.

Animals and other research organisms

Policy information about studies involving animals; ARRIVE guidelines recommended for reporting animal research, and Sex and Gender in Research

Laboratory animals

Female C57BL/6J mice (8-week-old) were purchased from Charles River Laboratories, Inc. (Kanagawa, Japan).
K5-cre mice were obtained from CARD R-BASE, Kumamoto University.
Sema3Afl/fl mice were purchased from Riken Bioresource Center (RBRC01106).

Wild animals

The study did not involve wild animals.

Reporting on sex

Female mice were used in this study.

Field-collected samples

No field-collected samples were used.

Ethics oversight

All mice were maintained under specific pathogen-free conditions and fed with autoclaved diet and water in the animal facilities at Tokushima University, they were treated in accordance with the National Institutes of Health Guide for the Care and Use of Laboratory Animals. All experimental procedures were approved by IACUC (No. T2019-51) and Institute for Genome Research (No.30-46) of Tokushima University.

Note that full information on the approval of the study protocol must also be provided in the manuscript.

Flow Cytometry

Plots

Confirm that:

☒ The axis labels state the marker and fluorochrome used (e.g. CD4-FITC).

☒ The axis scales are clearly visible. Include numbers along axes only for bottom left plot of group (a 'group' is an analysis of identical markers).

☒ All plots are contour plots with outliers or pseudocolor plots.

☒ A numerical value for number of cells or percentage (with statistics) is provided.

Methodology

Sample preparation	The ears of mice were split and cut, incubated with a solution of RPMI containing 1mg/ml DNase I (NIPPON GENE, Tokyo, Japan) and 1mg/ml collagenase (Worthington Biochemical, Lakewood, NJ, USA) 90 min at 37 ?. 1 x 10^6 cells of homogeneous cell suspension were incubated with Purified Rat Anti-Mouse CD16/CD32 for 5 minutes and stained with FITC anti-mouse CD11c, PE anti-mouse F4/80, APC-Cy™7 anti-mouse CD45, FITC anti-mouse CD3, PE/Cyanine7 anti-mouse CD4, PE anti-mouse CD8a, 7-AAD according to the recommended concentration. FITC Armenian Hamster IgG and PE Rat IgG2a, κ were used for isotype control. 1 x 106 Pam2.12 cells were stained with ReadidropTM Propidium Iodide (Bio-Rad Laboratories, Inc., CA, USA) according to the manufacturer instructions.
Instrument	BD FACSVerse™ (BDBiosciences, San Jose, CA, USA).
Software	BD FACSuite Software (BDBiosciences, San Jose, CA, USA).
Cell population abundance	Purity of the cells was between 95 and 99%.
Gating strategy	All experiments were gated first to identify lymphocytes, then exclude doublets using FSC-A vs FSC-H and SSC-A vs SSC-H. Cell surface markers were analyzed. The gating strategy was shown in Fig 4.

☐ Tick this box to confirm that a figure exemplifying the gating strategy is provided in the Supplementary Information.
